# Chemical and Structural Elucidation of Lignin and Cellulose Isolated Using DES from Bagasse Based on Alkaline and Hydrothermal Pretreatment

**DOI:** 10.3390/polym14142756

**Published:** 2022-07-06

**Authors:** Na Wang, Baoming Xu, Xinhui Wang, Jinyan Lang, Heng Zhang

**Affiliations:** 1 College of Marine Science and Biological Engineering, Qingdao University of Science and Technology, Qingdao 260412, China; wlalala21@163.com (N.W.); 14763738886@163.com (B.X.); wxhameq@163.com (X.W.); ljy17806248212@163.com (J.L.); 2 Guangdong Provincial Key Lab of Green Chemical Product Technology, Guangzhou 510640, China

**Keywords:** bagasse, DESs, separation, lignin

## Abstract

The separation of cellulose, hemicellulose, and lignin components using deep eutectic solvent, which is a green solvent, to obtain corresponding chemicals can realize the effective separation and high-value utilization of these components at low cost. In this study, we used waste biomass sugarcane bagasse as the raw material, choline chloride as the hydrogen bond acceptor, and lactic acid as the hydrogen bond donor to synthesize a deep eutectic solvent of choline chloride/lactic acid (L-DES) and treated sugarcane bagasse pretreated by alkali or hydrothermal methods to separate cellulose, hemicellulose, and lignin. In addition, we comparatively studied the effect of different pretreatment methods on lignin removal by DES and found that the lignin removal rate by L-DES after alkaline pretreatment was significantly higher than that after hydrothermal pretreatment, and the mechanism of action causing this difference is discussed.

## 1. Introduction

Finding an economical and efficient development pathway is important to achieve the high-value utilization of biomass resources. Data crop residues account for up to 54% of waste biomass resources [1]. Among the residues, sugarcane bagasse, which is produced abundantly, yields approximately 540 million tons worldwide every year [2].

Sugarcane bagasse is rich in cellulose, which can be used as a raw material for pulp and paper making, and its rich hemicellulose content is also conducive to the realization of various industries, such as xylose and xylitol; more than 200 value-added chemicals can be produced by refining lignocellulosic biomass [3], such as ethanol, levulinic acid, furan, sorbitol, xylitol, glycerol, and their derivatives [4]. Therefore, the prospects for the utilization of sugarcane bagasse as a waste biomass resource are very promising.

Deep eutectic solvent (DES) is an environmentally friendly green solvent that can solubilize lignin and increase the hydrophilicity of lignocellulose, thus increasing the glycation rate of lignocellulose. Hou et al. [5] found that the ester bond of the linkage between lignin and hemicellulose was broken from the infrared of lignocellulose treated with DESs, the hydrogen bond of the linkage between hemicellulose and cellulose was broken, and hemicellulose was hydrolyzed into oligosaccharides dissolved in DESs. Many studies have shown that DESs can be used as an effective solvent for the lignin separation of bagasse lignin, cellulose, and hemicellulose fractions, with a lignin removal of up to 81% [6,7,8,9,10].

**Figure 1:**
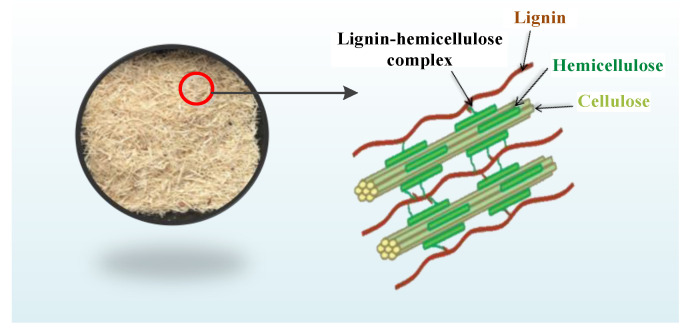
Three-dimensional structure of cellulose, hemicellulose, and lignin in bagasse.

As shown in Figure 1, hemicellulose acts as a binder to closely interpenetrate lignin and cellulose. Hemicellulose and lignin have chemical connections, and hydrogen bonding connections and Van der Waals forces exist between them and cellulose. Therefore, separating the three kinds of material while removing the hemicellulose is difficult and complicated. In addition, the glycosides bond of hemicellulose breaks under acidic conditions, leading to the degradation of hemicellulose into oligosaccharides [11]. Given that DES is acidic, the separation efficiency and purity of lignin can be further improved if the hemicellulose is pre-separated to improve the implementability of the DES separation of lignin and avoid the difficulty of separating hemicellulose and lignin by turning them into small molecules after acid hydrolysis.

Hemicellulose glycans can be dissolved in alkaline solution or hydrolyzed by dilute acid. The commonly used methods for hemicellulose separation include dilute acid hydrolysis, alkali extraction, hydrothermal extraction, and organic solvent extraction. Considering the concept of green process flow, the alkaline and hydrothermal hemicellulose extraction methods were selected and achieved highly satisfactory results [12,13].

However, the current study used DESs to remove lignin from sugarcane bagasse, and the products still retained hemicellulose, which did not report the effect of the pretreatment by alkali and hydrothermal methods on the subsequent lignin separation, regarding whether it would favor the lignin solubilization, or on the lignin structure.

In order to further improve the lignin removal rate and achieve effective separation of the components, therefore, a two-step treatment method was used in this study. Cellulase lignin (CEL), which has few structural alterations of lignin [14], was extracted from sugarcane bagasse feedstock as a representative of the total lignin in lignocellulosic feedstock, and choline chloride was used as the hydrogen bond acceptor and lactic acid as the hydrogen bond donor to synthesize the deep eutectic solvent of choline chloride/lactic acid (L-DES), which had good lignin removal and was essentially insoluble in cellulose in the previous experiments. The sugarcane bagasse pretreated by hydrothermal process and then treated by L-DES was hydrothermal lignin (HL), and after alkali pretreatment and L-DES treatment, the crude lignocellulosic was alkali lignin (AL). The structural changes of crude lignin HL and AL were compared and analyzed, and the differences in the mechanisms of lignin separation between the alkaline and hydrothermal pretreatments were also compared.

## 2. Materials and Method

### 2.1. Materials

Choline chloride, sodium acetate and hydrochloric acid were purchased from Sinopharm Chemical Reagent Co., Ltd. (Shanghai, China). Lactic acid was obtained from Shanghai Rinn Technology Development Co., Ltd. (Shanghai, China). Cellulase and dioxane were purchased from Shanghai Rinn Reagent Co., Ltd. (Shanghai, China). All the above reagents are analytically pure.

### 2.2. Experimental Methods

(1) Synthesis of L-DES

The sugarcane bagasse feedstock was dried and de-watered for 24 h before the experiment, and a molar ratio of choline chloride:lactic acid (1:9) was placed in a flask, heated, and stirred with N_2_ at 80 °C. The reaction was carried out for 1–2 h. When a uniform and transparent solution was obtained, the reaction was allowed to go on for another 20 min, further stabilizing the formed solution, and then the reaction was stopped. Finally, L-DES was obtained.

(2) Separation method of hemicellulose, cellulose, and lignin

1. Alkali separation of sugarcane bagasse hemicellulose

After benzene-alcohol extraction, bagasse was weighed, reacted in the alkali solution at a high temperature for a period of time, and then kept warm. After holding, the extract was poured into a beaker, the flask was washed three times with deionized water, and the washing solution was poured into the beaker.

The filtrate and the residue were separated by suction filtration. The residue was washed with deionized water until neutral, and the cake residue (bagasse after alkaline extraction, residue AB for short) was dried to a constant weight and used for the subsequent separation experiments. The filtrate (filtrate and the washing solution for cleaning the residue) was adjusted to a pH of approximately 5.5 using glacial acetic acid, left to stand, and then centrifuged. The precipitate was dried to obtain hemicellulose A. After the supernatant was concentrated by rotary evaporation, thrice the volume of 95% ethanol was added and allowed to stand for 2 h. The precipitate was washed with 70% ethanol after suction filtration and dried to obtain hemicellulose B (crude xylan sample). The supernatant was concentrated and dried to obtain alcohol-soluble lignin.

2. Hydrothermal separation of sugarcane bagasse hemicellulose

The bagasse, after benzene-alcohol extraction, was weighed and insulated for a period of time in a hydrothermal autoclave. After the insulation reaction, the hydrothermal autoclave was left to stand overnight under natural conditions and cooled to room temperature. The extract was poured into a beaker, the hydrothermal autoclave was washed three times with deionized water, and the washing solution was poured into the beaker.

The filtrate and residue were separated by suction filtration. After washing the residue several times with deionized water, the filter cake residue (bagasse after hydrothermal extraction, residue HB for short) was dried to a constant weight and used for the subsequent separation experiments. The filtrate (filtrate and the washing solution for cleaning the residue) was concentrated by distillation, and the hemicellulose was precipitated with thrice the volume of 95% ethanol. The experimental study showed that the hemicellulose yield was the highest when three times the volume of ethanol was used. Finally, the precipitate was centrifuged and freeze-dried to obtain a crude hemicellulose product, which was weighed to calculate the hemicellulose removal rate.

3. DES separation of sugarcane bagasse lignin

The dried residue obtained from the above two pretreatment methods was wetted with water (to reduce the lignin softening temperature) and reacted with L-DES at a high temperature for a certain time. After the reaction, the reaction solution was poured into a beaker, the four-necked flask was washed three times with anhydrous ethanol, and the washing solution was poured into the beaker.

**Figure 2:**
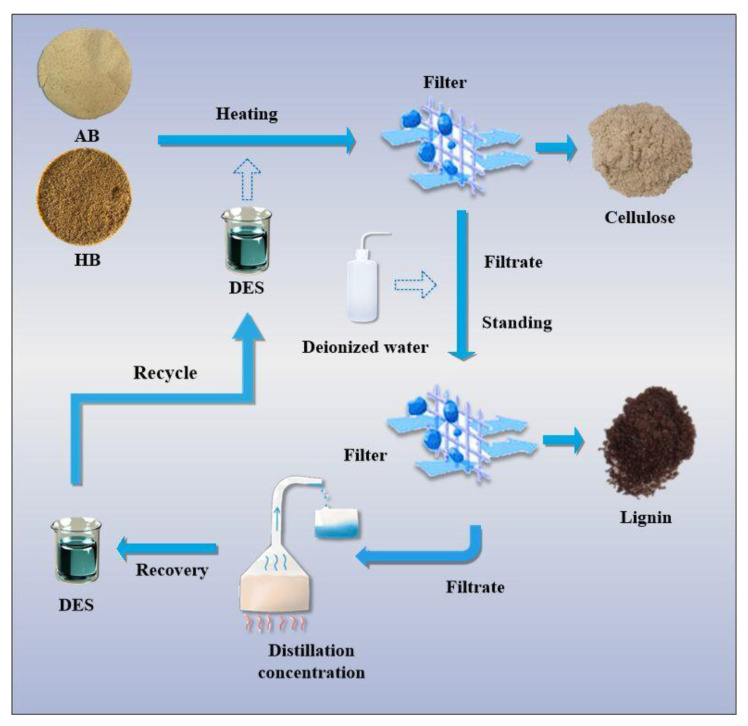
Flow chart of DES separation of lignin and cellulose.

The filtrate and the residue were separated by suction filtration, and the residue was washed with anhydrous ethanol for neutralization and dried in an oven to a constant weight to obtain crude cellulose. The filtrate was diluted with a large amount of deionized water and left to stratify, filtered to obtain a crude lignin solid, and dried to a constant weight and weighed to record the mass of crude cellulose and lignin. The filtrate was distilled from a large amount of anhydrous ethanol and deionized water using a rotary evaporator to obtain a small amount of viscous liquid, which was collected and recovered to obtain the DES for reuse. A flow chart is shown in Figure 2.

(3) Separation and extraction of cellulolytic enzyme lignin (CEL)

An enzymatic hydrolysis reaction was carried out by adding cellulase and acetic acid-sodium acetate buffer to the ground bagasse powder [15,16]. The precipitated residue after the enzymatic treatment was washed several times with acetic acid-sodium acetate buffer and deionized water and then freeze-dried. The residue was extracted using 96% aqueous dioxane solution, and the supernatant was concentrated by evaporation [16]. The concentrated solution was left to stand for precipitation with a hydrochloric acid solution, and the CEL precipitated out.

### 2.3. Characterization of Cellulose and Lignin

A small amount of dried bagasse, bagasse AB and HB after the respective hydrothermal and alkaline pre-extractions, cellulose AC and HC, and lignin AL and HL, AC which were separated after L-DES treatment, were compressed into tablets using KBr. Infrared measurements were taken using a VECTOR22 Fourier transform infrared spectrometer (Bruker Corporation, Ettlingen, Germany), with a scanning range of 4000–400 cm^−1^, 60 scan times, a resolution of 4 cm^−1^, and a signal-to-noise ratio of 55,000:1.

Crude cellulose AC and HC and lignin AL and HL were examined by scanning electron microscopy (SEM) using a Regulus 8100 SEM (Japan Electronics Co., Ltd., Tokyo, Japan) after hydrothermal and alkaline pre-extractions and L-DES treatment to observe the difference between the crude cellulose and lignin obtained by hydrothermal and alkaline extractions. The differences between crude cellulose and lignin were observed.

A TG209F1 thermogravimetric analyzer (Bruker Corporation, Ettlingen, Germany) was used to test the residues (AB and HB) obtained after the hydrothermal and alkaline pre-extractions, respectively, and the thermogravimetric behavior of crude cellulose AC and HC treated by L-DES was analyzed. A sample of approximately 10 mg was weighed, the temperature was increased from 25 °C to 700 °C at a rate of 10 °C/min under N_2_ protection, and the ventilation rate was 25 mL/min.

We accurately weighed 5 mg of cellulase lignin and the lignin samples AL and HL obtained by dissolving and separating with L-DES after alkaline and hydrothermal extractions, respectively, and set them in 5-mL centrifuge tubes. The pipette drew 5 mL of the chromatographically pure tetrahydrofuran (THF) to dissolve the lignin, and the centrifuge tube was placed in an ultrasonic wave for 2 h to dissolve the lignin and prepare the solution to be tested. A standard sample of polystyrene with a molecular weight of 6.9 × 10^4^ g/mol was used to calibrate the existing molecular weight standard curve, and the supernatant was aspirated into the sample with an injection needle for analysis. Gel permeation chromatography (GPC, Agilent, Santa Clara, CA, USA) was performed on a small molecular weight column tandem with a chromatographically pure THF eluent and a differential refractive index detector. The molecular weights of the lignin samples were calculated from the standard curve of the standard samples.

An elemental analyzer (Vario EL III, Langenselbold, Hesse, Germany) was used to determine the C, H, O, and N in bagasse and crude lignin AL and HL, and the results are all in mass fraction.

## 3. Results and Discussion

### 3.1. Comparison of Lignin Removal Rates by Hydrothermal Extraction Method and Alkali Extraction Method

**Figure 3:**
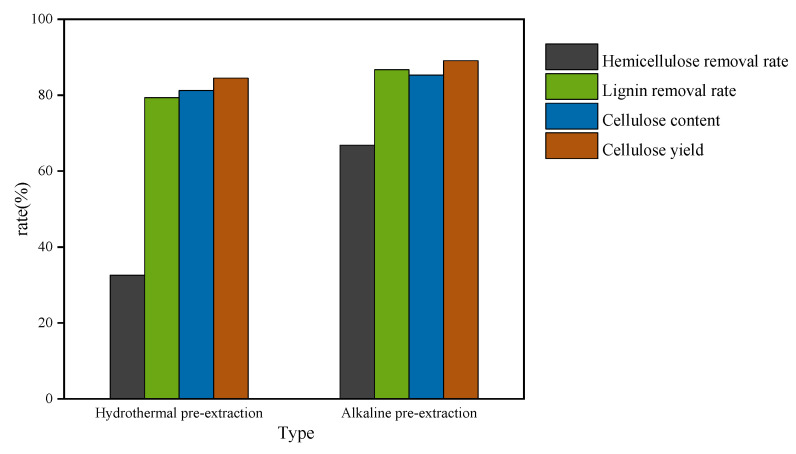
Effect of hydrothermal or alkaline pretreatment on the removal rate of lignin and hemicellulose, cellulose content and yield.

After the hydrothermal and alkali extractions, the lignin was removed by L-DES treatment, and the effects of the two methods on the lignin removal rate and the cellulose content and yield are shown in Figure 3.

Figure 3 shows a comparison of the test indicators, such as the lignin removal rate, under the two pretreatment methods. Under the best experimental conditions: solvent L-DES, bagasse: DES = 1:25, temperature of 110 °C, 12 h, alkali pretreated hemicellulose removal rate of 66.84%, lignin removal rate of 86.7%, and respective cellulose content and yield of 85.3% and 89.1%. The removal rate of hemicellulose by hydrothermal pretreatment was 32.57%, the removal rate of lignin was 79.6%, and the respective cellulose content and yield rate were 81.2% and 84.5%. The comparison shows that the removal rate of lignin after alkaline pretreatment was higher than that after hydrothermal pretreatment because under the action of alkaline, new phenolic hydroxyl (phenoxy anion) could be derived from lignin, which is conducive to the dissolution of lignin due to the hydrophilic property of phenolic hydroxyl. In addition, according to the literature on the treatment of bagasse with DESs, L-DES is used to dissolve lignin in the residue after alkaline pretreatment. This work could obtain the highest removal rate of lignin according to the existing studies on lignin removal from bagasse with DESs treatment [6].

### 3.2. Structure Analysis of Crude Lignin and Cellulose by Infrared Spectroscopy

**Figure 4:**
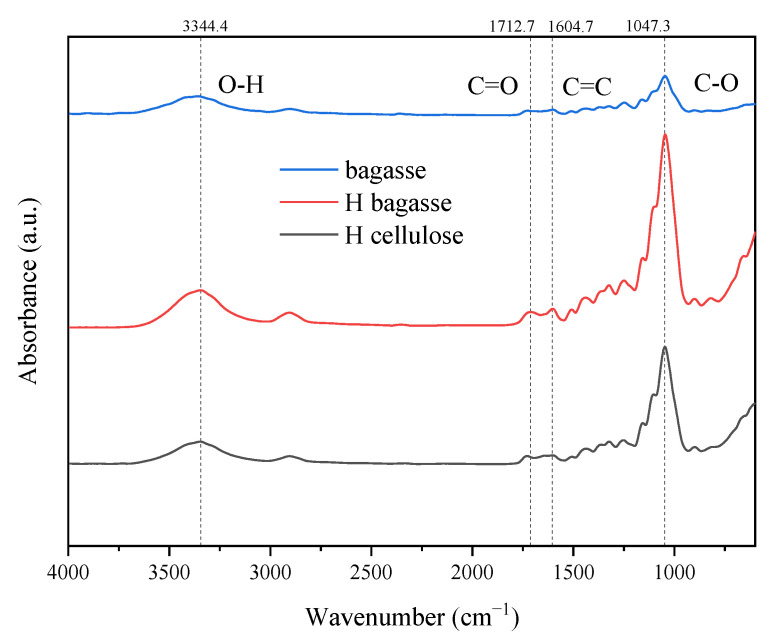
Infrared spectra of bagasse, HB and HC.

**Figure 5:**
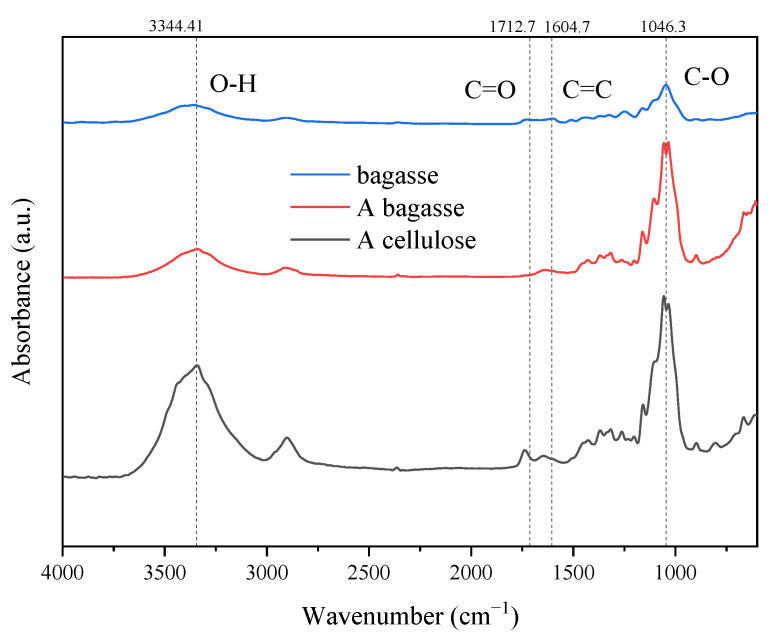
Infrared spectra of bagasse, bagasse after alkaline treatment (AB) and crude cellulose after L-DES treatment (AC).

The infrared spectra of bagasse, bagasse after hydrothermal and alkali pre-extraction treatments, and bagasse after L-DES treatment are shown in Figure 4 and Figure 5.

The infrared spectra of bagasse, HB, and HC are shown in Figure 4. The broad peak at 3344 cm^−1^ is the -OH stretching vibration peak, and the -OH content is highest in cellulose HC. The -OH content increased relatively due to the removal of hemicellulose. The stretching vibration peak of C-H is at 2904 cm^−1^, including the -CH and -CH_2_ of the saturated hydrocarbon group and the -CH_3_ of the methoxy group on the benzene ring. The bending vibration peak of C-H at 1330 cm^−1^, 825 cm^−1^ is the C-H vibration peak linked with the benzene ring [17], 1604 and 1510 cm^−1^ are the skeleton vibration absorption peaks of benzene ring, 1242 cm^−1^ is the C-O vibration absorption peak of the phenolic hydroxyl group, and 1045 and 1101 cm^−1^ are the respective bending and stretching vibration peaks of the ether bond on the benzene ring, which represent the characteristic absorption peaks of lignin. The absorbance of these characteristic peaks in cellulose HC decreased significantly, indicating that lignin was removed in large quantities. However, the absorbance of these characteristic peaks in residue HB increased slightly because the lignin content in residue HB increased relatively with the removal of hemicellulose, so the characteristic peaks were enhanced slightly.

The strong absorption peak at 1101 cm^−1^ is also the stretching vibration peak of the alicyclic ether C-O-C, which is the alicyclic ether bond in cellulose and hemicellulose five- and six-carbon sugars. The intensity of the characteristic peak decreased obviously with the removal of lignin and hemicellulose. The C=O vibration absorption peak of hemicellulose is at 1712 cm^−1^, which is the characteristic absorption peak of C=O on the acetyl group [18]. The absorbance of C=O in residue HB did not decrease obviously because the hemicellulose removal rate through the hydrothermal extraction method in this research was not high. The characteristic peak intensity in cellulose HC is weak because hemicellulose was dissolved in DESs. The β-1, 4-glycosidic bond of cellulose is located at 819 cm^−1^. After L-DES treatment, a part of the glycosidic bond of cellulose was broken, so its strength decreased slightly.

Figure 5 shows the IR spectra of sugarcane bagasse, bagasse AB after alkali treatment, and the crude cellulose AC obtained after L-DES treatment. The characteristic peak of lignin weakened from the bagasse to residue AB to cellulose AC, indicating that the alkali extraction removed part of the lignin from the bagasse, while the L-DES solvent removed most of the lignin from the bagasse. The intensity of the C=O vibrational absorption peak of hemicellulose xylose at 1735 cm^−1^ showed a similar general trend, the hemicellulose in sugarcane bagasse was gradually removed by alkali extraction and the L-DES.

The peak at 3344 cm^−1^ is assigned to the -OH stretching of cellulose, and the peak at 2904 cm^−1^ is assigned to the C-H vibration of cellulose. The strength of the -OH and C-H stretching vibration peaks were all weakened significantly after the alkali extraction and the L-DES treatment, suggesting that the hydroxyl content in cellulose gradually decreased. The β-1, 4-glycosidic bond of cellulose is located at 819 cm^−1^, the glycosidic bond in the residue AB was broken and its strength was significantly decreased, indicating that the alkali caused some degree of damage to the cellulose structure. The -OH stretching vibration bands blue-shifted from the bagasse to residue AB, and the wavelength from 3344 cm^−1^ to 3371 cm^−1^; from residue AB to cellulose AC, the wavelength shifted from 3371 cm^−1^ to 3404 cm^−1^; the bending vibration peaks of C-H red-shifted from the bagasse to residue AB, and the wavelength from 1321 cm^−1^ to 1316 cm^−1^; from residue AB to cellulose AC, the wavelength shifted from 1316 cm^−1^ to 1313 cm^−1^. These data suggest that the intermolecular hydrogen bonds of cellulose suffered from the disruption of alkali and L-DES, which also inevitably led to structural changes in the crystalline region of cellulose [19].

**Figure 6:**
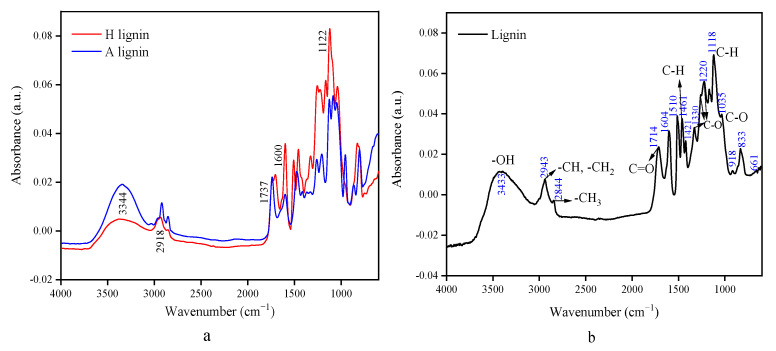
Infrared spectra of alkaline lignin AL and hydrothermal lignin HL separated by L-DES after alkaline pre-extraction and hydrothermal pre-extraction (**a**) and cellulase lignin (**b**).

Figure 6a shows the spectra of AL and HL, and Figure 6b shows the spectra of cellulase lignin.

Cellulase lignin is extracted by solubilizing cellulose and hemicellulose using cellulase enzymes, and its structure is the most similar to the original natural lignin structure. Figure 6(b) shows the peaks of the telescopic vibrations of the phenolic and aliphatic hydroxyl groups of lignin at 3433 cm^−1^, the C-H telescopic vibrations of -CH and -CH_2_ at 2943 cm^−1^, the C-H telescopic vibrations of -CH_3_ at 2844 cm^−1^, and the C-H asymmetric bending vibrations of methoxy at 1461 cm^−1^ and 1421 cm^−1^, and the fingerprint region at 833 cm^−1^ is the C-H vibration peak of the benzene ring linkage. The conjugated C=O stretching vibration peak in lignin is located at 1714 cm^−1^ [20], which is the C=O bond in the ketone or the aldehyde group of lignin. The basic structural units in bagasse lignin are the lilac- and guaiac-based phenylpropane units and a small amount of the p-hydroxyphenyl phenylpropane unit. Among the phenylpropane units, the lilac-based phenylpropane unit is the most abundant, and the C-O deformation vibration of this phenylpropane unit is located at 1330 cm^−1^. The C-H in-plane bending vibration of the phenylpropane unit is located at 1118 cm^−1^; 1263, 1220, 1220, and 1035 cm^−1^ are the peaks of the C-O stretching vibrations in lignin; 1220 cm^−1^ is the C-O stretching vibration in the guaiacyl phenylpropane unit [21]; 1035 cm^−1^ is the C-O stretching vibration of the secondary alcohol or fatty ether structure [22]; and 1604 and 1510 cm^−1^ are the absorption peaks of the benzene ring skeleton vibrations [23].

The comparison of Figure 6a, b indicates that the hydroxyl content of HL decreased slightly, which may be attributed to the reaction of the lignin hydroxyl group with the chloride ion of L-DES, and the reaction reached the high softening temperature of lignin with partial methoxy removal. The intensity of the bending vibrational peak within the C-H plane of the AL decreased significantly, showing that the phenyl propane unit was damaged. Moreover, the -OH stretching vibration peak in lignin blue-shifted and the C-H asymmetric bending vibration red-shifted, suggesting that the hydrogen bonding structure of alkali lignin was changed by the influence of alkali and the strength of hydrogen bonding interaction slightly decreased. The red-shift of the C-O stretching vibration peak in alkali lignin demonstrates that the electron cloud density between its C-O bonds is lower than that of the original lignin, the bond strength constant decreased, the bond strength weakened, and the vibration frequency decreased. This phenomenon indicates that alkali caused some C-O bonds to break.

### 3.3. Morphology Analysis and Comparison of Crude Cellulose AC and HC

**Figure 7:**
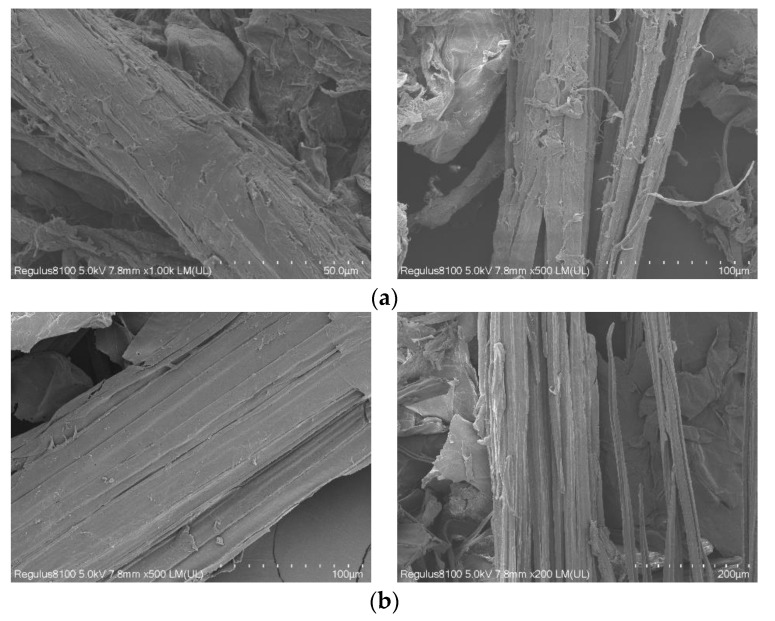
Scanning electron micrographs of crude cellulose AC (**a**) and crude cellulose HC (**b**).

After detecting the crude cellulose AC and HC by SEM, the difference between the crude cellulose HC and AC obtained by hydrothermal and alkaline extractions was observed. The scanning electron micrographs of crude cellulose HC and AC are shown in Figure 7.

Figure 7a shows crude cellulose AC amplified by 1000 and 500 times; Figure 7b shows crude cellulose HC amplified by 500 and 200 times. Unlike that of crude cellulose AC, the surface of crude cellulose HC is relatively smooth. In addition, the difference between the structures of crude cellulose HC and AC is not obvious, and their diameters are also similar. The fiber bundles were subjected to L-DES. L-DES dissolved the hemicellulose as adhesive and degraded it to small molecule oligosaccharides. In addition, the ether bonds of lignin were broken and degraded into small molecule guaiac-based compounds, leading to the dispersion of the fiber bundles.

### 3.4. Analysis of Thermogravimetric Behavior

**Figure 8:**
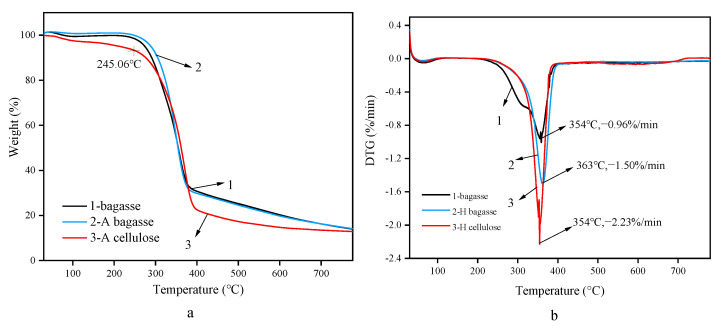
Thermogravimetric curves TG (**a**) and DTG (**b**) of two stages of hydrothermal extraction.

The thermogravimetric behaviors of residues AB and HB obtained after bagasse, hydrothermal and alkaline pre-extractions and those of crude cellulose AC and HC after L-DES treatment are shown in Figure 8.

(Among them, 1-bagasse; 2-bagasse HB after hydrothermal extraction; 3-cellulose HC after hydrothermal extraction).

The comparison of the thermogravimetric curves of bagasse, residue HB, and crude cellulose HC is shown in Figure 8. The three curves between 260 °C and 380 °C show a significant decline in mass. The mass loss rate is very high, reaching the maximum rate of weightlessness between 350 °C and 365 °C. The maximum rates of weightlessness on the three curves are 0.96%/min, 1.50%/min, and 2.23%/min, respectively.

The thermogravimetric processes of bagasse and cellulose can be divided into three periods; they are turned into solid residue and gases, such as CH_4_ and CO, through thermal decomposition. The first period is mostly the process of losing free water. During this process, the weight of residue HB and cellulose HC was basically unchanged, while the weight loss of bagasse was 2.38%. Bagasse had the highest free water content. The second period is the main period for the thermal decomposition of bagasse and cellulose. Bagasse had the lowest initial decomposition temperature, which was 260 °C, because the unstable hemicellulose and cellulose were decomposed under the influence of the high temperature and pressure. Therefore, the HB structure of the remaining cellulose is relatively stable and has a higher decomposition temperature. The maximum weight loss rate was also achieved in this period, and the maximum weight loss rate temperature is in the range of 350 °C to 365 °C. The weight loss of bagasse was approximately 70%, the weight loss of residual HB was 73%, and the weight loss of cellulose HC was 78%, suggesting that cellulose HC has the highest carbon content because it has the highest cellulose content. Bagasse, residue HB, and cellulose HC entered the third weightless period from approximately 377 °C to approximately 775 °C. Cellulose HC had the lowest quality of raw material residue, which was 7.83%, while those of residue HB and bagasse were 14.96% and 13.86%, respectively. It was observed that the HC particle size of the cellulose treated by DES decreased in the experiment and the specific surface and heated areas increased, facilitating the decomposition. In addition, the ash content of cellulose is less than that of lignin. After DESs treatment, the lignin content significantly decreased, so the residual content of cellulose HC was much lower than residue HB. The leftover content of residual HB is higher than that of bagasse because at the same weight, the lignin content in residual HB and the non-decomposed part is higher, so the residual quality is also higher.

**Figure 9:**
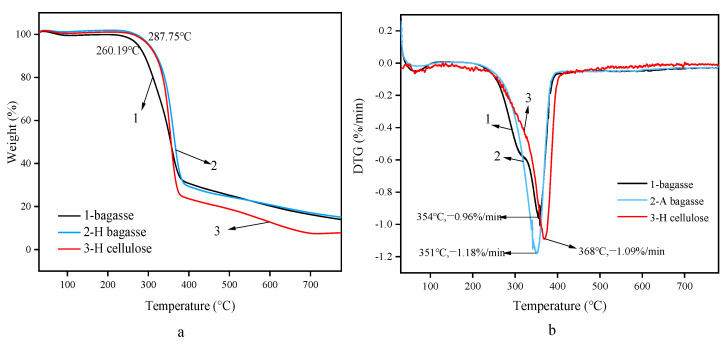
Thermogravimetric curves TG (**a**) and DTG (**b**) of two stages of alkaline extraction.

The thermogravimetric curves of bagasse, residue AB, and crude cellulose AC are compared in Figure 9.

(Thereinto,1-bagasse; 2-bagasse extracted by alkali AB; 3-alkali cellulose Ac).

In the 240 °C to 390 °C range, the three curves exhibit a distinct descent and a very high mass loss rate. The respective maximum weight loss rates were 0.96%/min, 1.18%/min, and 1.09%/min in the 350 °C to 370 °C range.

The first period of thermogravimetry is mainly the loss of free water. In this process, the weight loss of bagasse and residual AB was very small, while the weight loss of cellulose AC was relatively high (6.31%), probably because cellulose absorbs water and expands under alkaline conditions, thereby increasing the free water content. The initial decomposition temperature of the three was approximately 245 °C, and they entered the second interval from 245 °C. The pyrolysis rates of bagasse and cellulose were basically the same, and the maximum weight loss rate was in the 350 °C to 370 °C range. The weight loss of bagasse was approximately 70%; that of residue AB was 71%; and that of cellulose AC was 73%, suggesting that cellulose AC had the highest carbon content. The third weightlessness period was from approximately 390 °C and terminated at about 775 °C. The weights of the raw material residues of the three are similar; the residue weights of bagasse and residual AC are the same weight, that is, 14.05%, and that of cellulose AC was slightly lower, that is, 12.93%. The residue quality of cellulose AC was higher than that of cellulose HC because the metal salt ions promoted coke formation.

### 3.5. Analysis of Lignin Molecular Weight

The results of the AL and HL samples obtained by CEL and alkaline and hydrothermal extractions followed by dissolution and separation with L-DES were measured by GPC, as shown in the table below. The number-average molecular weight (Mn), heavy-average molecular weight (Mw), Z-average molecular weight (Mz), Z+1-average molecular weight (Mz1), and polymerization distribution width index D of lignin were calculated from the calibration curve measured by the GPC system. Then, the heavy-average number-average ratio (Mw/Mn) and the Z-average heavy-average ratio Mz/Mw were calculated.

As shown in the Table 1, both lignin average molecular masses Mn obtained using L-DES treatment are slightly lower than that of CEL, ranging from 1900 to 3500. The molecular weight of lignin AL is lower than that of lignin HL because alkali disrupted the structure of lignin and the various ether bonds in lignin broke under the action of alkali, resulting in the decrease in polymerization and lignin molecular weight. The lower the numbers of aromatic ether bonds and condensed structures in lignin are, the lower the molecular weight is [24].

High-temperature water extraction had little effect on the lignin structure, and the molecular weight of HL was not as large as the average molecular weight of natural lignin mainly because the reaction conditions were not mild enough. The solvent L-DES separation of lignin has a relatively strong effect on the lignin structure because the strong hydrogen bonding of lactic acid leads to a strong lignin degradation reaction [25], so that the molecular weight of the lignin obtained is usually small. However, the effect of L-DES on the structure of lignin is significantly smaller than that of lye.

Among the lignin separation and extraction methods, the difference in molecular weight between ground wood lignin (MWL) and CEL and the original lignin [26] is the smallest, although MWL and CEL have much lower molecular weights than the original lignin. For the Mw fraction, the molecular weight can be as high as 20,000 in order of magnitude, which is actually difficult to reach for the extracted lignin molecular weight. The polymerization distribution width index (D) can reflect the polydispersity of lignin and indicates the inhomogeneity of the relative molecular mass of lignin. The polymerization distribution width index of lignin was significantly lower after L-DES treatment, indicating that lignin formed subunits with a relatively small molecular weight and a more uniform structure through the degradation process [20]. The polymerization distribution width index (D) of lignin AL was 1.23, which is slightly higher than that of lignin HL of 1.15, indicating a highly uneven distribution of its lignin relative molecular mass. This phenomenon is due to the uneven destruction of the lignin structure by the alkali solution; the lignin structure on the surface was more damaged than that inside, which also led to the uneven distribution of its relative molecular masses. However, the polymerization distribution width index (D) values of 1.15–1.23 are still generally low, and the lignin AL and HL relative molecular masses are still distributed relatively uniformly.

### 3.6. Elemental Analysis

The results of the determination of the C, H, N, and O contents in crude cellulose and lignin using an elemental analyzer are shown in the Table 2.

As shown in Table 2, the carbon content of lignin AL was significantly higher than that of lignin HL, indicating that more lignin was extracted by alkaline pre-extraction. The result suggests that alkaline pre-extraction can dissolve lignin better than hydrothermal pre-extraction to obtain a higher rate of lignin extraction. This is also consistent with the TG analysis results that the molecular weight of lignin AL is low, and the residue mass of lignin AL is high. Both lignin AL and HL have a higher C content but a slightly lower O content than CEL because lignin is rich in methoxy, carbonyl, and hydroxyl groups and ether, carbon-oxygen, carbon-carbon, and other connecting bonds. The lower O content may indicate that reactions occurred, such as methoxy shedding and ether bond breaking. The H content of lignin AL was higher than that of lignin HL, probably because more phenolic hydroxyl groups were generated by the alkali method and the shedding of some of the carbonyl groups and breaking of some of the C=C bonds.

Given that lignin is an aromatic polymer with high unsaturation, the C content of all three lignin was higher and the H content was slightly lower than those of the sugarcane bagasse feedstock. The carbohydrates in the sugarcane bagasse feedstock contain many hydroxyl and alicyclic ether structures and are less unsaturated than lignin, so the O and H contents are higher than those of lignin.

## 4. Conclusions

Structural analysis revealed that both alkaline and hydrothermal pretreatments had good lignin removal efficiency. The hydroxyl content of hydrothermal lignin HL decreased slightly, the hydrogen bond strength of alkaline lignin AL weakened, and the phenyl propane unit was damaged, which indicated that both alkaline and hydrothermal pretreatments had some effects on the structure of lignin, while the alkaline pretreatment caused more effects on the structure of lignin. Alkali and L-DES caused some degree of damage to the β-1, 4 glycosidic bonds of cellulose, with alkali causing more damage to cellulose.

The L-DES-treated cellulose had an increased specific surface area, which is more easily decomposed with less residue mass and has a decreased lignin content. Compared with CEL, the polymerization distribution width index of lignin was significantly lower after L-DES treatment, indicating that lignin formed subunits with a relatively small molecular weight and a homogeneous structure through the degradation reaction.

## Figures and Tables

**Table 1:** Molecular weight of alkaline and hydrothermal lignin.

Sample	Mn	Mw	Mz	Mz1	Mw/Mn	Mz/Mw
CEL	3793	5319	6819	7942	1.40232	1.28200
AL	1904	2347	2782	3279	1.23267	1.18534
HL	3448	3973	4620	5346	1.15207	1.16294

**Table 2:** Elemental composition of alkaline and hydrothermal lignin.

Sample	C/%	H/%	O/%	N/%
Bagasse	52.32	8.51	33.71	1.26
CEL	62.10	6.32	27.94	1.15
AL	65.47	6.45	25.83	1.17
HL	63.53	6.09	27.41	1.05

## Data Availability

Data sharing not applicable.
